# Participatory Action Research as a Driver for Health Promotion and Prevention: A Co-creation Process Between Professionals and Citizens in a Deprived Neighbourhood in the Hague

**DOI:** 10.5334/ijic.7560

**Published:** 2023-11-29

**Authors:** Wilma van der Vlegel-Brouwer, Madelon Eelderink, Jet Bussemaker

**Affiliations:** 1 Seven Senses Institute, Nieuwegein, The Netherlands; 2 LUMC/Leiden University, The Hague, The Netherlands; 3 Utrecht University, The Netherlands; 4 Institute of Public Administration, Health Campus The Hague, The Netherlands

**Keywords:** health promotion, health prevention, Participatory Action Research, health inequalities

## Abstract

**Introduction:**

Ignited by the persistent health inequalities many cities and neighbourhoods, the ‘Healthy and Happy The Hague’ network in the Netherlands wanted to gain insight in how prevention and health promotion could become successful in one deprived neighbourhood, Moerwijk.

**Methods:**

The cycle of Look-Think-Act of Participatory Action Research was used in which both citizens and professionals got involved from the start. Besides interviews, field notes were analysed, visualised and discussed in several rounds of focus groups.

**Results:**

Thematic analysis yielded seven themes: Healthy Eating and Exercise, Healthy Money, Healthy Mind, Healthy Relationships, Growing up healthy, Healthy Environment and Healthy Collaboration. During sessions around combination of themes, eight initiatives were co-created by citizens and professionals together, improving the feeling of ownership and interconnectedness.

**Discussion and conclusion:**

This PAR sheds a light on the mismatch between the system world’s solutions for individuals and the living world’s needs for solutions for the collective. Findings provides a better insight into the social, political, and cultural mechanisms and processes that influence clustering and interaction of health conditions. PAR is a promising process of citizens and professionals working together is an excellent way to learn about the conditions under which people experience health inequalities, and how to combat these inequalities.

## Introduction

Societies face major variation in health experience and in many countries public health is challenged to improve health and reduce health inequalities [1]. Health inequalities are the systematic, avoidable and unethical differences in health outcomes that can be observed between populations, between social groups within the same population or as a gradient across a population ranked by social position [2]. Although *reduced inequalities* is the tenth of the United Nations’ Sustainable Development Goals since 2015 to be achieved by 2030, inequalities seem persistent. Evidence of these inequalities strongly supports social causes and the COVID-19 pandemic, climate catastrophes and economic crisis seem to increase existing inequalities [1,3]. In the Netherlands, health – both in terms of life-expectancy and experienced years in good health – has increased as the result of policy interventions. Health inequalities, however, have hardly declined and have even increased in some respects [4,5]. Many cities are unable to reduce health inequalities, despite local integrated approaches. There is an increasing attention for applying Participatory Action Research (PAR) in the search for a more integrated community-based approach aimed at reducing local health inequalities [6].

PAR has its roots in both Lewin’s approach of action research and participatory research [7,8,9]. PAR involves the participation and leadership of those people experiencing issues, who take action to produce emancipatory social change, through conducting systematic research to generate new knowledge. PAR is an approach that contributes to community development by emphasising participation and action by members of communities affected by that research [10]. It seeks to understand the world by trying to change it collaboratively, recognising the existence of a plurality of knowledges throughout the entire process. PAR contributes to social learning, mutual understanding of each other’s perspectives, a sense of empowerment and the co-creation and realisation of action plans by all stakeholders, which are included throughout the entire process [9]. Building blocks for PAR are: building relationships; establishing working practices; establishing a common understanding of the issue; observing, gathering and generating materials; collaborative analysis; and planning and taking action [11]. This emergent process is based on reflection, data collection, and action, in which learning and change are embedded in both the processes and outcomes of the research [12]. One of the underlying values of PAR is to recognise the existence of a plurality of knowledges and to maximize participation. PAR differs from most other approaches to public health research, which predominantly presuppose an objective reality that can be measured, analysed and predicted by suitably qualified individuals [8,13]. PAR enables a so called community-up approach, i.e. citizens, professionals and other stakeholders involved at every level to take action, in this study on improving health and reducing health inequities as co-researches in a collective process of co-creating and co-learning by applying methods that actively involves stakeholders from the start [9].

Health sciences, including public health, is strongly influenced by the values and assumptions of evidence-based medicine and the associated ontological and epistemological underpinnings. Recently however, there is a growing attention for experiential knowledge through increasing the participation of the people affected by health issues in research and policy development [14,15]. The change from hierarchical approaches to health is also evident in policy and practice increasing focus on people-centeredness. The positive effects of citizen participation, like more efficient use of scarce resources, increased self-resilience of citizens, and improved well-being, are promising [16,17,18].

PAR as a research approach introduces a different way to shed light on community profiling, as a social research method which involves building up a picture of the nature, needs and resources of a locality or community, with the active participation of its members, with the aim of co-creating initiatives to address the issues unearthed [19].

Community profiling starts with clear characteristics of the neighbourhood. Merged open access data of the government, municipalities, and the National Institute for Public Health and Environment (RIVM) and Statistics Netherlands (CBS), indeed show that Moerwijk, the focus neighbourhood of this PAR, is known to have 21.000 citizens of which 25% are native residents, 62% of the citizens are single and the average income is €18.200. 62% of the citizens between 18 and 65 years has an average to high risk for anxiety or depression [20].

In the city of The Hague, a local city network of the Municipality, care and welfare partners, The Leiden University-Campus The Hague, citizens’ initiatives, knowledge institutes and health insurers, called Healthy and Happy The Hague, agreed that a neighbourhood-oriented approach such as PAR is necessary to work sustainably on the health and happiness of residents.

Although the parties involved agreed in principle upon a neighbourhood approach, in which the life journey of the citizen is a key concept, practice was problematic. The start of the programme showed a lack of insight among professionals into citizens’ viewpoints on the programme. Three years funding from the Ministry of Health, Welfare and Sport enabled parties involved to improve health prevention and health promotion. The approach of Participatory Action Research (PAR) was used between February 2021 and September 2021, with the objective to gain insight how prevention and health promotion could become successful in deprived neighbourhoods. The aim of this first cycle of PAR was to bridge the gap between the system world of organisations and professionals on the one hand and the daily life experience of citizens on the other hand while at the same time facilitate professionals and citizens to co-research the situation of health inequalities and to co-create solutions that aim to improve health.

## Research methods

This PAR addressed four main questions:

1. What are the needs of the citizens of Moerwijk with regard to health prevention and health promotion?
2. Which factors contribute to the current experiences of staying healthy?
3. Which resources to promote healthy living and a healthy lifestyle are available in the neighbourhood?
4. What shift is needed for professionals, policy and legislation to enable processes of co-creation with citizens?

This PAR was based on the PAR routine of Look – Think – Act [21]. In the Look-phase we observed what energised the group and collected the different perspectives on the problem and the possible directions for solutions. In the Think-phase we gave back the results of our data analysis and started a dialogue and reflection between participants. In the Act-phase we facilitated co-creation of plans with members of the group, which they then executed together. Below, the methods used in each of these stages is described in more detail.

First, citizens were recruited by purposeful sampling of parents and children, persons with mental health care needs or chronic diseases and older adults. These groups resembled the target groups of “Happy and Healthy The Hague”. Additionally, professionals from health and social services working in this neighbourhood were recruited.

As a first step in our PAR approach for citizens, the ‘tangerine-method’ was applied – a method the first author developed to get a first impression of how people perceive their health: on a tangerine with glued eyes on it, citizens were asked to draw a mouth indicating how healthy they felt, as a conversation starter [22]. Next, if people gave consent, more in depth interviews were held. Data were gathered through observations and interviews during meetings in community centers, schoolyards, at squares and on sport fields and through focus group sessions. Participants, both citizens and professionals, were invited to share their views, dreams and solutions. Notes were taken and recorded in a log. Data of narratives during focus groups were recorded by a second PAR practitioner, who wrote down the observations and stories during the sessions. These data were added to the log for analysis.

Data from the interviews were analysed using Green and Thorogood’s thematic analysis [23]. We familiarised ourselves with the data by asking ourselves “what is this?” and “what is this about?” Using Google Sheets, participants’ perceived current situations, desired situations, needs, assets, solutions and values (“what is this?”) were identified to formulate a so-called argumentation lines [9]. For each argumentation line the main theme was identified (“what is this about”?). Data from in total 60 in depth interviews (23 professionals and 37 citizens) and logbook notes of additional meetings during seven fieldwork days (one foodbank, two schoolyards, two community centers and one sports field and one playground) were analysed to identify patterns of possible overarching themes. For each theme a visualisation was made, representing all perspectives. In addition, an overall visualisation was made showing the interconnectedness of all themes. Every participant who provided us with their name and contact details was invited for the focus group meetings.

During the focus group sessions, findings from the Look phase – as presented in the visualisations- were presented to participants, and a dialogue about those findings was facilitated, allowing participants to further express themselves and add missing information. In following focus group sessions, participants were invited to brainstorm about possible solutions and co-create the best suitable action plans that they could then realise together. In the following section, the findings are presented for each phase of the PAR process, further clarifying how findings led to the chosen methods for the next phase.

Lastly, the entire PAR process and it’s outcomes is reflected upon among the authors of this paper, leading to what is described in our discussion, conclusion and recommendation section.

## Findings

First, our study-approach focused on gathering perspectives. These perspectives were analysed and an overall visualisation was made, elucidating these perspectives. This thematic analysis yielded seven themes. Healthy Diet and Exercise, Healthy Money, Healthy Mind, Healthy Relationships, and Growing up healthy reflect the day-to-day issues. The themes Healthy Environment and Healthy Collaboration are more overarching in influence all the other themes, especially as the system world and the living world often do not seem to reach each other.

The section below describes 1) the content of the main themes, 2) co-creation of initiatives and impact.

### Main Themes

1. *Healthy Diet & Exercise* is about food (security), knowledge of healthy eating and cooking (with a small budget) and exercise. Citizens want to do something with their lifestyle. However, the available information about healthy eating or the proposed lifestyle change often does not match their needs. Care professionals’ focus is on individual responsibility. Different (eating) patterns, limited budget or other complex life matters that require attention are listed as barriers.
*“Prevention, exercise, obesity (…) – what we do as separate organisations is a drop in the ocean.” – professional*
*“Health and healthy lifestyle is a kind of luxury version of life for me and I am actually surviving. So I have not reached that level yet, if you don’t mind.” – citizen*
2. *Healthy Money* is about financial stability and sufficient resources. The citizens shared their need for a future perspective, also economically, opportunities to develop one’s talent and more long-term guidance.
*“We have 80 euros per week for our family (3 children) which is quite nice. Only you really cannot do anything else. Extra expenses get me in trouble, like medicines.” – citizen*
*“People have to survive – stress doesn’t contribute to thinking about how to get out of welfare benefits.” – professional*
3. *Healthy Mind* is about mental health, which is necessary to take good care of yourself. Stress and mental health problems are seen as barriers to make healthy choices. Citizens, including many young people, experience mental problems and recognise these among others. Citizens experience that many professionals have insufficient knowledge in this area.
*“Many girls feel the pressure to perform. This affects the mental health of the girls. In this neighbourhood, the parent-child conversation about this is a problem.” – professional*
*“There is a huge stigma attached to mental health problems. Often well-intentioned advice, including from professionals, has not helped me. I wish I could be someone I missed myself”. – citizen*
4. *Healthy Relationships* is about social cohesion in the neighbourhood, about citizens’ networks, but also about (the quality of) parent-child relationships. The network of many residents however, appears to be limited, often bound to their own cultural background, and the problem of parent-child relationships is transferred to problems in the street such as nuisance and violence. Often, insufficient Dutch proficiency is a major barrier in developing relationships. In addition, many professionals indicate they do not know the neighbourhood well and that they are at a distance from the citizens.
*“Asking for help is difficult. Often, when this happens people are already in serious trouble – that is mopping with the tap open. Then suddenly there is all kinds of help, mental health care, debt counseling, a few more agencies – that is too much for parents.” – citizen*
*“That parallel society I talked about … I’ve seen it for a long time…Segregation is the problem. You can spend millions on initiatives, but if people go to their own clubs, it makes no sense. They adhere to their own norms and values.” – professional*
5. *Growing up Healthy* is about upbringing and the social, cultural and healthy living environment in which children grow up. It is one of the most frequently mentioned themes. There are great concerns in the neighbourhood about letting young people grow up in an unsafe environment. Many parents wish to leave the neighbourhood, but feel trapped. Boredom, a lack of or undesired activities for young people seem to amplify other problems. Citizens and professionals claim that many young people run into narcotics or criminal activities.
*“The mothers are the most important target group for prevention – they are raising the new generation.” – citizen*
*“Kids are not always raised well and young people often hang out on the streets. This creates an unsafe feeling for older citizens.” – professionals*
6. *Healthy Living Environment* is about the design of the neighbourhood, housing or homelessness, greenery, waste and safety. Housing and safety cause a lot of stress and have a major impact on mental well-being. There is a lot of nuisance from residents who are under the influence of alcohol or drugs, there is parking nuisance and nuisance from litter and rats. Citizens say everything is intertwined and it feels like a perpetual cycle.
*“There is also little to do in this neigbourhood. This is a major problem for children and young people. The only thing we have is a community center.” – professional*
*“There are a lot of addicts in the neighbourhood – how do we prevent this in the next generation – what are the children now getting as an example?” – citizen*
7. *Healthy Collaboration* is about how system world and daily experience of citizens are clashing and how interventions of the system world are experienced by citizens. The lack of collaboration between professional organisations and the limited connections of professionals from health – and social services with the communities was mentioned as one of the big barriers. Making connections with citizens is challenging for professionals and many professionals reported to be unfamiliar with the neighbourhood. There are concerns about not knowing each other, coordination problems, frameworks of organisations being barriers, competition and lack of trust. Professionals and citizens shared stories about getting lost in the multitude of projects and interventions in the neighourhood and consequently, problems in referring someone to the right care attendant. Many projects are short term due to time-limited funding. Capacity problems and the rapid changes in staff cause trust issues. Citizens are seldomly involved in the development of plans and do not feel an equal partner in the implementation. Citizens’ initiatives are often unknown to professionals and collaboration between professional services and citizen initiatives is often lacking.
*“What you get is that every organisation will do their own trick [provide their own service]. Those young people will receive help from many sides for a short period of time and no one is integrating those services.” – citizen*
*“Who really are we, as organisations? Shouldn’t we, as professionals, also become a kind of sharing community? What do we actually share with each other?” – professional*

Many themes are interwoven and influence each other. For example, citizens cannot think of a healthy lifestyle (Healthy Diet & Exercise) when they experience high stress levels (Healthy Mind) which is often caused by external factors such as financial challenges (Healthy Money) or unhealthy living conditions (Healthy Living Environment), such as intimidating youngsters in the streets. The latter is mainly caused by boredom and/or poor upbringing (Growing Up Healthy). PAR-participants claim that you can only grow up healthy if your living environment is healthy. Especially a healthy environment is considered key to break the perpetual cycle and is seen as a prerequisite for meeting each other, working together and being able to play outside.

### Co-creation of initiatives and impact

The insights of the themes and overall visualisation were shared during focus group meetings and reflected upon with all involved participants. Next, participants were invited around themes and additional visualisations around themes were reflected upon in a first round of focus groups (Table 1). In a second or third round of focus groups co-creation of initiatives was facilitated by the PAR practitioners, as well as discussions about the ways to connect citizens’ experiences with the evidence based solutions of professionals. Interviews were transcribed and analysed by thematic analysis. Findings and solutions were discussed in focus groups, which addressed one or more themes. In subsequent focus groups, the co-creation of action plans was facilitated based upon these reflections between all the stakeholders.

**Table 1:** Themes and focus group meetings.


THEME	FOCUS GROUPS (SUBGROUPS/SESSIONS)	CITIZENS (PER SESSION/TOTAL)	PROFESSIONALS (PER SESSION/TOTAL)	FACILITATORS (PER SESSION)

Healthy Diet and Exercise & Growing up Healthy	***6*** *(2 /3)*	*N = 6/ N = 36*	*N = 2/ N = 12*	*2*

Mental Health & Healthy Relationships	***2*** *(1/2)*	*N = 4/ N = 8*	*N = 2 / N = 4*	*1*

Healthy Collaboration	***2*** *(1/2)*	*N = 4*	*N = 10/ N = 20*	*2*

Healthy Environment	*1 (1/1)*	*N = 15*	*N = 4*	*1*

Healthy Lifestyle	*1 (1/1)*	*N = 15*	*N = 3*	*1*


**Figure 1:**
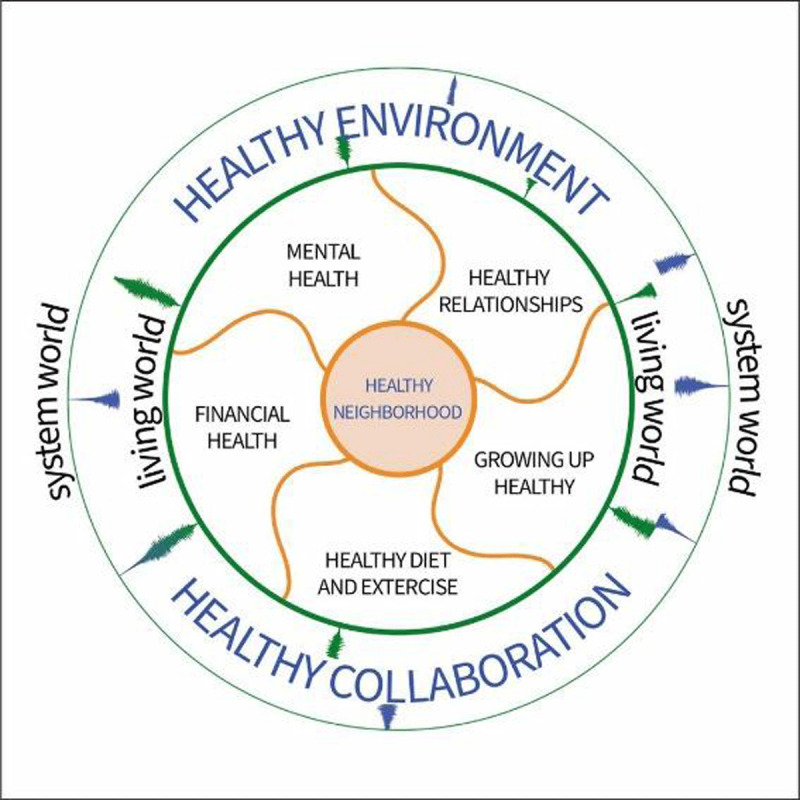
Visualisation of themes and interrelatedness.

During the focus groups, citizens and professionals reflected upon the overall visualisation of the seven themes (Figure 1), and on a more detailed visualisation (not shown in this paper) of each theme that was addressed in several specific focus groups.

Participants brainstormed about several directions for solutions, and co-created appropriate action plans on the prioritised ideas and initiatives. Eleven focus group meetings were organised with citizens and professionals around a combination of themes, as listed in Table 1, such as Healthy Mind & Healthy Relationships, Healthy Diet & Exercise and Growing up Healthy, or more overarching focus groups on themes of Healthy Living Environment. Upon request from the community, one additional focus group was organised around healthy lifestyle. A couple of focus groups specifically addressed Healthy Collaboration of professionals. In every focus group it was apparent that all themes were intertwined and should be addressed in conjunction with each other.

Citizens stated they first needed to work on their relationships in the neighbourhood and their mental health before they could work on other health-aspects. The *Healthy Living Environment* theme appeared to be very important in all conversations. There is a great desire to work together towards a situation where children can play outside safely again and where neighbours can informally meet each other. The need emerged to create more awareness on outdoor space policies and how a sustainable ecosystem could be developed around the entire food process. Regarding *Healthy Collaboration*, both within citizens’ associations and between professional organisations, dialogues started in which ownership, frameworks, findability and sustainability were shaped together and trust could be restored. It became apparent that citizens focus on solutions aimed at the collective of the community, and professionals mainly focus on solutions for the individual, leading to different needs and views on desired outcomes.

During PAR, citizens – from their living world perspective – and professionals – from their system world perspective – learned from each other’s viewpoints and became more connected. Solutions were created together that moved away from the dominant evidence-based lifestyle approaches. Working together as professionals and citizens resulted in a total of 8 initiatives. Two mother groups started, with the focus on building social networks. An initiative started on creating safe spaces in the neighbourhood, especially for mental wellbeing. A group of women started swimming lessons especially for women. Others started to develop a cooking book with the neighbourhood, aiming at cooking on a budget, and an initiative started for the sharing of ideas, cloths, food and other goods in the entire neigbourhood. During the monitioring-phase of this PAR, citizens’ confidence increased as they contributed to these initiatives on community groups

*“I feel the same confidence to speak up as when I was in my twenties”. -citizen*

Citizens started to speak up more, felt more empowered, and started other initiatives by themselves, like breakfast groups for women. They talked to the policymakers at the municipality for instance to arrange swimming lessons for women with and without an islamic background. However, we also saw the tendency of professionals to take over from citizens after the start of some of the initiatives or to say they already had the solution for the problem. For example, in the process developing a cooking-on-a-budget book for and with the neighbourhood, professionals disrupted the process by just collecting recipes from citizens in order to develop a book themselves. This discouraged the citizens, and hampered the continuance of the initiatives, as their focus was to strengthen their social networks.

## Discussion

This first cycle of PAR created the first ripples for change. The PAR approach specifically aims at a non-linear process of joint learning on a shared complex problem which is investigated with various stakeholders [24]. After PAR participants reflected on the results of the Look phase and based upon their positive energy, they co-created the best fitting interventions. Also in PAR not every problem encountered is addressed in the interventions, but a start is made to achieve sustainable impact by addressing those issues everyone is enthusiastic for. The impact of a PAR starts during its process and continues after a PAR cycle is finished. It contains many forms of change that occur with, within and for those who are engaging in PAR. In our PAR, observed changes encompassed increased empowerment, confidence, feeling listened to, more social contacts, feeling part of a group, more safety, more well-being, feeling appreciated and improved self-rated health.

The impact of this PAR reaches beyond the initiatives themselves, as the interconnectedness between people has improved. In a neighbourhood where many citizens come and go, attention has to be paid to long-term engagement of all actors, as the process of building trust and social networks takes time. Although every PAR participant was invited to the focus groups, not everyone was able or willing to attend. This stresses the challenges for participating in and building long-term partnerships. Due to time constraints only a sample of citizens and professional could be reached. PAR is, however, a promising process of professionals and citizens working together, community-up, to address health inequalities, building trust and creating ownership. Whereas it is not advisable to copy paste initiatives to other neighbourhoods, the methodology of PAR, although time consuming at first, is easily transferable due to its flexible character. Although this PAR focused on the community level as the most desirable level of action, attention should be paid to all levels and domains of influence. For example, when authority actors within organisations facilitate their professionals in working together with communities, initiatives can be co-created that specifically address citizens’ needs in that specific moment in time. Therefore, a shift is needed towards flexible, open and collaborative practices in which professionals feel more space to differentiate from traditional work routines when they feel they need to.

PAR is an excellent way to learn about the conditions under which people experience health inequalities and their perspectives on how to create a health and social care system that is tailored to their needs [25]. This PAR contributes to understanding and unpacking the root causes of health inequalities and igniting change in this regard. These outcomes are aligned with several recent publications of The Council of Public Health & Society, an independent advisory body to the government and parliament in the Netherlands [26,27]. Freudenberg and Tsui (2014) state that PAR is the only empirical method available to public health that allows such wide-ranging assessments of complex realities and policy and political engagement. Being conscious about the root causes challenges existing political power structures and contributes to a process of linking local health concerns to structural causes, rather than seeing it as the product of individual behaviour [28]. Although challenging for PAR teams, PAR addresses this so called ‘local trap’ and enables actors, with unequal capacities and powers, from the entire hierarchical ladder to co-create solutions where their talents and possibilities complement each other [29].

Through PAR, community profiling can be done by facilitating local actors to identify community needs and -later in the PAR process- co-create action plans to address those needs that will help reduce health inequalities [19]. Especially in neighbourhoods with complex and wicked interrelated problems, PAR provides the opportunity to explore with citizens and professionals of different domains together, which domain(s) of life (e.g. Figure 1) should be addressed first or simultaneously and in what way. This provides the opportunity to reframe or reinvent evidence-based solutions as those solution often do only take one of the aspects of the lifeworld into account and leave other interrelated aspects out of scope.

Many (evidence-based) interventions in health care and social services focus on the individual level of health promotion, which is often only on a part of complex situations, and on situations where there is already loss of health and well-being. Citizens in deprived neighbourhoods with complex social problems, such as Moerwijk, do not experience an integrated approach that addresses their intertwined issues and problems. The different conceptions of health by frontline professionals hampers the necessary interprofessional approach [30]. In addition, the one-sided and individual approach does not contribute to breaking the perpetual cycle of health inequalities. During this PAR, citizens shed a light on the mismatch between the system world’s solutions for individuals and the living world’s needs for solutions for the collective, aiming at health promotion, like improving the environment they live in, and solutions that acknowledge them as independent, autonomous and valuable in themselves. This mismatch causes: 1) citizens feeling unheard, 2) organisations putting more and more efforts into promoting their own solutions, 3) a decline in health instead of better health, 4) themes of creating a community together, strengthening mental wellbeing as well as improving safety by social interactions remaining underdeveloped.

There were several challenges during this process. At first there is a tendency among professionals as well as citizens to focus on what is wrong, not on what is strong, which leaves the capacities, talents and assets of the citizens underexposed and undervalued [31,32]. Therefore, we had to encourage PAR participants to also share assets of the community. Secondly, professionals experience organisational and professional boundaries in speaking up and doing the right thing when it deviates from their usual work routines. They need more time and space to think ‘outside’ the box and to feel free to work between the different domains of health and social care. As the professionals’ tendency is to regard citizens in those neighbourhoods as less equipped to define their situation properly and to take a more prominent role in initiatives, the danger lies in moving towards traditional ways of working and taking over from citizens, resulting in disrupting the development of ownership of citizens. Up until now, system values seem to dominate the lifeworld and expert knowledge seems to be more valued than expressed emotions and narratives of citizens [33].

## Conclusion

This PAR provides a better insight into the social, political, and cultural mechanisms and processes that influence clustering and interaction of health conditions in a neighbourhood in the Hague. However, the focus on the community level as the most desirable level of action is questionable if it is not combined with the other levels and domains of influence. The PAR approach needs not only to be embraced at the community level, but also at the organisational and policy level. This would not only transform the way organisations work together, but also change the paradigm of working *for* citizens to working *with* citizens. Sharing power and control is still challenging. Power inequalities also affect PAR teams and communities, and have to be understood as one of the root causes for continuing health inequalities. It is worth investigating how the system world can create the ideal conditions for the community-up approach by means of PAR that can lead to more impact in society on health inequalities. Current organisational interest and policies insufficiently provide the space and attitude needed to work together with communities and together as collaborative organisations. The ‘Healthy and Happy The Hague’ movement still has a lot of underused political clout to sway policymakers and other key stakeholders towards this new way of working together to bring about change and long-term engagement with communities and all stakeholders involved, on the road of reducing health inequalities. Reiterative cycles of PAR are necessary to accomplish structural and impactful change. Citizens as well as professionals worry most about the new generation growing up healthy. This could provide common ground to develop a new way of working together.

## Recommendations

### Policy

For sustainable impact and to address health inequalities, at a policy level PAR should be embraced as an approach in a collective effort aligning the goals of different organisations with the ideas of citizens. Public health and social service professionals and researchers should be offered training in participation and in navigating the tensions between policies, science and the lifeworld. During the process of co-creation, managers are recommended to give their professionals the space to deviate from work routines where necessary and question and alter current evidence-based approaches in collaboration with citizens. Long-term funding is needed for transdisciplinary collaboration and co-creative engagement.

### Practice

It is recommended to allow a PAR process to consist of several consecutive PAR cycles and incorporate multi-agencies and multi-levels to address structural causes of health inequalities. PAR needs follow up, as there is a tendency to fall back to evidence based practices that do not work, especially in neighbourhoods with low SES status. Consecutive PAR cycles help prevent that and can build on initial initiatives and strengthen the voices of citizens and street level professionals.

### Research

More research is needed on how organisations in the system world can work together with citizens in a community-up approach and thus create more impact on a societal level. Public health care research should move away from research focusing solely on understanding problems and include PAR minded approaches that focus on understanding problems while solving them at the same time.
